# A perspective on Oxford’s R21/Matrix-M™ malaria vaccine and the future of global eradication efforts

**DOI:** 10.1186/s12936-024-04846-w

**Published:** 2024-01-12

**Authors:** Nicholas Aderinto, Gbolahan Olatunji, Emmanuel Kokori, Sodeeq Sikirullahi, John Ehi Aboje, Rebecca Ebokondu Ojabo

**Affiliations:** 1 Department of Medicine and Surgery, Ladoke Akintola University Teaching Hospital, PMB 5000, Ogbomoso, Nigeria; 2 Department of Medicine and Surgery, University of Ilorin, Ilorin, Nigeria; 3 College of Health Sciences, Benue State University, Makurdi, Benue Nigeria; 4 Afe Babalola University College of Medicine and Health Sciences, Ado-Ekiti, Nigeria

**Keywords:** Malaria, Vaccine, R21/Matrix-M, WHO, Eradication, Global Health

## Abstract

Malaria affects millions of lives annually, particularly in tropical and subtropical regions. Despite being largely preventable, 2021 witnessed 247 million infections and over 600,000 deaths across 85 countries. In the ongoing battle against malaria, a promising development has emerged with the endorsement by the World Health Organization (WHO) of the R21/Matrix-M^™^ Malaria Vaccine. Developed through a collaboration between the University of Oxford and Novavax, this vaccine has demonstrated remarkable efficacy, reaching 77% effectiveness in Phase 2 clinical trials. It is designed to be low-dose, cost-effective, and accessible, with approval for use in children under three years old. This perspective paper critically examines the R21/Matrix-M malaria vaccine, its development, potential impact on global malaria eradication efforts, and the challenges and opportunities it presents.

## Background

Malaria affects millions of lives yearly, severely impacting tropical and subtropical regions [1]. Despite being largely preventable, in 2021 alone, an estimated 247 million people were infected with malaria across 85 countries, resulting in over 600,000 deaths [2]. In the ongoing global struggle against malaria, a promising development has emerged. The World Health Organization (WHO) recently endorsed the R21/Matrix-M^™^ Malaria Vaccine for preventing malaria across vulnerable age groups. This endorsement followed guidance from the Strategic Advisory Group of Experts on Immunization (SAGE) and the Malaria Policy Advisory Group (MPAG) [3]. Developed through a partnership between the University of Oxford and the Serum Institute of India, a major supplier of vaccines to Africa, together with Novavax, a large biotechnology company which supplied the adjuvant, the R21/Matrix-M vaccine builds on the partial success of the RTS,S/AS01 malaria vaccine demonstrated in clinical trials. It represents a significant advancement in providing enhanced protection against malaria [4].

Notably, phase 3 clinical trials have shown the R21/Matrix-M 12-month Vaccine Efficacy (VE) to be 75% in locations characterized by seasonal variations, while it registered at 68% in standard locations [1]. The VE against multiple clinical malaria episodes exhibited a comparable pattern, with rates of 75% at the seasonal sites and 67% at the standard sites, marking a substantial step forward in the battle against this disease [1]. Designed to be low-dose, cost-effective, and readily accessible, this vaccine holds immense potential for malaria-endemic countries in tropical regions. It has also received approval for use in children under 3 years old, who are at the highest risk of malaria-related fatalities [5, 6]. This perspective paper aims to analyse the R21/Matrix-M Malaria Vaccine, its development process, its potential impact on global malaria eradication efforts, and the challenges and opportunities it presents.

## The breakthrough vaccine

Studies have shown that the R21/Matrix-M vaccine exhibits remarkable efficacy and safety in Phase II trials, including among children who received a booster dose of R21/Matrix-M 1 year after completing a primary three-dose regimen [7]. Initially focused on inducing high-level T-cell responses against pre-erythrocytic liver-stage malaria antigens, the vaccine's scope has recently expanded to include the induction of high-level antibodies against the sporozoite stage of the life cycle [1]. A distinguishing feature of this vaccine is the inclusion of Novavax’s Matrix-M, a saponin-based adjuvant known for enhancing immune responses, resulting in greater effectiveness and durability. Matrix-M stimulates the recruitment of antigen-presenting cells at the injection site and improves antigen presentation in local lymph nodes. This technology has previously proven successful in Novavax's COVID-19 vaccine and plays a critical role in developing other vaccines in the pipeline [6]. Notably, the R21/Matrix-M vaccine boasts higher efficacy than previous malaria vaccines, offering up to 77% protection against the malaria parasite in clinical trials. It is among the most promising candidates for an effective malaria vaccine [3, 8].

In October 2021, the WHO recommended the RTS, S/AS01 malaria vaccine for preventing *P. falciparum* malaria in regions with moderate to high transmission, particularly among children, but this vaccine provides only partial immunity against malaria in children [5]. What sets the R21/Matrix-M vaccine apart is its efficacy among children, who are often the most vulnerable to malaria [7]. This enhances its potential to have a broad and enduring impact on malaria control [9]. However, there is an observed decline in VE among older children [1]. Vaccine-induced antibodies targeting the conserved central NANP repeat sequence of the circumsporozoite protein exhibited a robust correlation with VE. The 5–17-month age group demonstrated significantly higher antibody titers than 18–36-month-olds (p < 0.0001). Specifically, when comparing the two age groups, VE was significantly higher in the younger age group (78%) than in the older age group (70%). This was observed in both seasonal and standard sites: in the seasonal sites, efficacy in the younger age group was 79% and at standard sites, 75%. Importantly, the difference in VE between the two age groups was statistically significant (p < 0.05), emphasizing the importance of developing malaria vaccines with high efficacy across all age groups. Further studies are warranted to explore the factors contributing to the decline in VE in older children.

Furthermore, despite the encouraging results observed in the published clinical trials, it is important to acknowledge concerns regarding VE in both seasonal and standard sites. While the recently released Phase III results did not identify significant differences in VE between seasonal and standard sites, a discernible trend toward higher efficacy in seasonal sites was noted [1]. Specifically, over 12 months, VE for the time leading up to the initial clinical malaria episode was 75% in the seasonal sites and 68% in the standard sites [1]. Similarly, VE against multiple clinical malaria episodes exhibited comparable figures: 75% in the seasonal sites and 67% in the standard sites. A parallel decline in efficacy over the initial year of follow-up was observed at both seasonal and standard sites. Notably, at the seasonal sites, the introduction of a booster dose demonstrated sustained efficacy for up to 18 months: VE was 74% for the time to the first clinical malaria episode and 72% against multiple clinical malaria episodes. This suggests a potential role for timing and consideration that the VE dynamics might differ if R21 were implemented non-seasonally as part of routine childhood vaccination programmes.

It is also important to note that the original RTS,S trial did not include chemoprevention in its design, setting it apart from the recent R21 Phase III trial [10]. While the authors of the R21 trial adjusted for the confounding factor of chemoprevention and found no discernible impact on protective associations, a lingering debate questions whether such adjustments truly replicate the conditions of an efficacy trial where chemoprevention was absent [1]. This is crucial in interpreting the malaria vaccine trial outcomes.

Nonetheless, this innovation represents a significant leap towards reducing the mortality and morbidity caused by this disease. It may pave the way for the global eradication of this preventable yet devastating illness. According to the WHO, the R21 vaccine has been proven safe in clinical trials [3], and like other new vaccines, safety monitoring will continue.

## Future directions and prospects

The future of malaria control hinges on recognizing that malaria is a complex and multi-dimensional problem [11]. While the R21/Matrix-M^™^ Malaria Vaccine is a promising tool, it cannot work in isolation. Malaria control should encompass a range of interventions, including vector control, prompt diagnosis and treatment, community education, and infrastructure development. Moreover, as malaria is endemic in diverse settings, the approach must be adaptable and context-specific. What works in one region may not be suitable for another. Integrating these various strategies in a coordinated manner at the local, national, and international levels is essential. Therefore, tailoring malaria interventions, including vaccine deployment, based on local data and context is imperative. Local factors such as mosquito species, climate, and healthcare infrastructure can significantly influence the effectiveness of interventions. Robust research and data collection efforts are essential to inform decision-making. Furthermore, community engagement is vital. Local communities must be involved in the design and implementation of control measures. This enhances the effectiveness of interventions and fosters community ownership and sustainability.

The inclusion of vaccines in comprehensive malaria control plans signifies a paradigm shift. Historically, malaria control primarily relied on vector control and treatment [12]. The introduction of vaccines represents a significant advancement. These vaccines serve as a means to protect individuals and as tools to reduce overall malaria transmission in endemic areas. Integration also requires robust surveillance systems to monitor vaccine impact and malaria prevalence. This data-driven approach ensures that vaccines are deployed strategically, targeting high-transmission areas with the most significant impact.

High-burden regions, especially in sub-Saharan Africa, demand particular attention [1]. These areas experience the greatest malaria burden, including the highest mortality rates among children under five. Deploying vaccines like the R21/Matrix-M^™^ in these regions can substantially reduce malaria-related morbidity and mortality. Equity in vaccine distribution is essential to ensure that vulnerable populations in high-burden regions have access to the same level of protection as those in lower-burden areas. Efficient distribution and administration are essential to ensure vaccines reach the intended recipients. Timely deployment and proper cold chain management are critical to maintain vaccine efficacy. Minimizing wastage is cost-effective and ensures that vaccines are used to their full potential. Furthermore, equitable access to vaccines is essential to prevent disparities in coverage. This requires addressing logistical challenges, infrastructure gaps, and supply chain issues.

## Conclusion

The endorsement by the WHO of the R21/Matrix-M malaria vaccine represents a significant step towards global malaria eradication. Its remarkable efficacy and ongoing scale-up efforts hold immense promise. However, it is essential to recognize that vaccines are not a standalone solution, but a critical addition to existing interventions. A multifaceted approach that combines vaccination with other proven measures will be key to successfully combating malaria and achieving long-term eradication.

## Data Availability

Data sharing is not applicable to this article as no datasets were generated or analysed during the current study.

## Abbreviations

VE: Vaccine Efficacy
WHO: World Health Organization
RTS,S/AS01: Malaria vaccine abbreviation
COVID-19: Coronavirus Disease 2019
R21/Matrix-M: Malaria vaccine abbreviation
